# Disease activity and damage in juvenile idiopathic arthritis: methotrexate era versus biologic era

**DOI:** 10.1186/s13075-019-1950-7

**Published:** 2019-07-08

**Authors:** Gabriella Giancane, Valentina Muratore, Valentina Marzetti, Neus Quilis, Belen Serrano Benavente, Francesca Bagnasco, Alessandra Alongi, Adele Civino, Lorenzo Quartulli, Alessandro Consolaro, Angelo Ravelli

**Affiliations:** 1 Dipartimento di Neuroscienze, Riabilitazione, Oftalmologia, Genetica e Scienze Materno-Infantili, Università degli Studi di Genova, Genoa, Italy; 2 Clinica Pediatrica e Reumatologia, IRCCS G. Gaslini, via G. Gaslini 5, 16147 Genoa, Italy; 3 Clinica Pediatrica, IRCCS Fondazione Policlinico San Matteo, Pavia, Italy; 4 Ospedale Vito Fazzi, Lecce, Italy; 5 UOC Pediatria, Azienda Ospedaliera Cardinale G. Panico, Tricase, Italy; 6 Sechenov First Moscow State Medical University, Moscow, Russian Federation

**Keywords:** Juvenile idiopathic arthritis, Disease activity, Disease damage, Long-term outcome, Methotrexate, Biologic agents

## Abstract

**Objective:**

To compare the long-term disease state, in terms of activity and damage, of children with juvenile idiopathic arthritis (JIA) who had their disease onset in methotrexate (MTX) or biologic eras.

**Methods:**

Patients were included in MTX or biologic era cohort depending on whether their disease presentation occurred before or after January 2000. All patients had disease duration ≥ 5 years and underwent a prospective cross-sectional assessment, which included measurement of disease activity and damage. Inactive disease (ID) and low disease activity (LDA) states were defined according to Wallace, JADAS10, and cJADAS10 criteria. Articular and extraarticular damage was assessed with the Juvenile Arthritis Damage Index (JADI).

**Results:**

MTX and biologic era cohorts included 239 and 269 patients, respectively. Patients were divided in the “functional phenotypes” of oligoarthritis and polyarthritis. At cross-sectional visit, patients in the biologic era cohort with either oligoarthritis or polyarthritis had consistently higher frequencies of ID and LDA by all criteria. The measurement of disease damage at cross-sectional visit revealed that the frequency of impairment of > 1 JADI-Articular items was higher in MTX than in biologic era cohort (17.6% versus 11% in oligoarthritis and 52.6% versus 21.8% in polyarthritis). Likewise, frequency of involvement of > 1 JADI-Extraarticular items was higher in the MTX than in the biologic era cohort (26.5% versus 16.2% in oligoarthritis and 31.4% versus 13.5% in polyarthritis).

**Conclusion:**

Our study provides evidence of the remarkable outcome improvement obtained with the recent therapeutic advance in JIA.

## Introduction

Over the past three decades, the management of juvenile idiopathic arthritis (JIA) has evolved considerably. Historically, the therapeutic advance in this disease has been marked by two major breakthroughs: the first was represented by the introduction of methotrexate (MTX) in the mid of the 1980s; the second, which took place in the 2000s, was brought by the marketing of biologic disease-modifying antirheumatic drugs (DMARDs). Owing to their profound impact on therapeutic approaches and disease prognosis, these two periods could be labeled as “MTX era” and “biologic era”, respectively [1, 2].

The efficacy of MTX in JIA was initially suggested by Truckenbrodt and co-workers in 1986 [3] and was then established in a US-former USSR collaborative randomized controlled trial (RCT) published in 1992 [4]. Based on these achievements, MTX became shortly the DMARD of choice in JIA. A subsequent multinational RCT conducted by the Paediatric Rheumatology International Trials Organization (PRINTO) concluded that the plateau of efficacy of MTX is reached with parenteral administration of 15 mg/m^2^ per week (the maximum weekly dose being 20 mg/m^2^) [5]. In the same epoch, the widespread use of MTX was paralleled by the growing popularity of intraarticular corticosteroid therapy [6]. This therapeutic procedure was initially used only for the knees, but was then applied to other joints and performed repeatedly [7, 8]. Several outcome studies published in the early 2000s documented improved outlook for children with JIA in the MTX era as compared to previous decades [9–11].

The first biologic DMARD studied in JIA was etanercept. Its effectiveness and acceptable safety profile were established in a RCT based on the withdrawal design, published in 2000, in patients with polyarticular-course JIA who were refractory or intolerant to MTX [12]. Over the following years, three other anti-tumor necrosis factor (TNF) agents (infliximab [13], adalimumab [14], and golimumab [15]) were tested in RCTs in patients with polyarticular course with JIA and a trial with another TNF-blocking agent (certolizumab pegol) is currently being performed [16]. In 2008, abatacept, another biologic DMARD with a different mechanism of action, was registered for use in the same disease subset [17]. Other RCTs established the effectiveness and tolerability of the IL-6 inhibitor tocilizumab [18, 19] and the anti-IL-1 agents anakinra [20], canakinumab [21], and rilonacept [22, 23] in systemic JIA. The sustained efficacy and high retention rate of most of these agents was demonstrated in long-term extension surveys of the original RCTs [24–26].

In recent years, the therapeutic aim has been increasingly moved toward the evaluation of novel strategies based on early aggressive interventions. Altogether, these approaches were found to lead to clinical inactive disease in a substantial proportion of patients with all phenotypes of JIA [27–30]. A further improvement in disease outcomes will likely be obtained with the implementation of the treat-to-target strategy [31]. After nearly two decades from the start of the biologic era, systematic analyses of patient series treated with contemporary therapies have shown a high frequency of attainment of inactive disease and satisfactory levels of physical function and quality of life [32–35]. However, the outcomes for children with JIA treated the biologic or MTX eras have seldom been compared. This question was addressed in the present study, which was aimed to compare the long-term status in terms of disease activity and damage of two cohorts of children with JIA, one with disease onset in the MTX era and one with disease onset in the biologic era.

## Methods

### Study design and patient selection

Based on the above-described timeline, we included in the MTX era cohort patients with disease onset between January 1986 and December 1999, and in the biologic era cohort patients with disease onset after January 2000.

Patients in the MTX era cohort and part of the patients in the biologic era cohort were taken from a previous cross-sectional study published by our group [11], which was aimed to evaluate the disease status of a consecutive sample of JIA patients with a minimum disease duration of 5 years who underwent a cross-sectional assessment between 2002 and 2006. This investigation enrolled 310 patients who had a disease duration of ≥ 5 years and a disease onset between December 1986 and December 2002. Patients in this study were placed in the MTX or biologic era cohort depending on whether they had their disease onset before or after January 2000, respectively.

An additional sample of patients with onset in the biologic era was enrolled in a subsequent prospective cross-sectional study, which included all consecutive patients meeting the International League of Associations for Rheumatology (ILAR) criteria for JIA [36] who were seen consecutively at the Istituto G. Gaslini of Genoa, Italy, between January 2015 and June 2017, had a disease duration of ≥ 5 years and a disease onset between January 2002 and June 2011.

Levels of disease activity and damage could be compared across patients included in the two studies as these two constructs were assessed using the same clinical instruments. Cross-sectional assessment was performed in all patients at the last follow-up visit.

Patients with enthesitis-related arthritis had been excluded from our previous cross-sectional study [11] and were, therefore, not included also in the subsequent prospective cross-sectional study.

All parents/guardians or patients (as appropriate) provided informed consent to participation in the study. The study protocol was approved by the Ethics Committee of the Istituto G. Gaslini, Genoa, Italy.

### Clinical assessment

At study visit, the following information was obtained for each patient by reviewing clinical charts: sex, age at disease onset, ILAR category, disease duration, age at study visit, ANA status, and previous and present therapies with synthetic and biologic DMARDs and systemic and intraarticular glucocorticoids.

The following clinical assessments were made by the attending pediatric rheumatologist: physician global assessment of overall disease activity, measured on a 21-numbered circle visual analog scale (VAS; where 0 = no activity and 10 = maximum activity), and count of joints with active arthritis, as described [37]. Laboratory tests included erythrocyte sedimentation rate (ESR) and C-reactive protein (CRP).

Prior to the study visit, a parent of each child was asked to make a global assessment of child’s wellbeing on a 21-numbered circle VAS (where 0 = very good and 10 = very poor) and to rate the intensity of child’s pain on a 21-numbered circle VAS (where 0 = no pain and 10 = very severe pain).

### Assessment of disease activity

The level of disease activity was computed by means of the Juvenile Arthritis Disease Activity Score 10 (JADAS10) [38] and its clinical (3-item) version, the cJADAS10 [39, 40]. Briefly, the JADAS10 is composed of the following four variables: (1) physician global assessment of overall disease activity, (2) parent global assessment of child’s wellbeing, (3) 10-joint reduced active joint count, and (4) ESR. The JADAS10 is calculated as the sum of the scores of its individual components, which yields a global score of 0–40. The cJADAS10 has the same structure of the JADAS10, but lacks the acute phase reactant. Its score ranges from 0 to 30.

Inactive disease (ID) was defined by Wallace, JADAS10, and cJADAS10 criteria [40–42]. By Wallace criteria, ID is established if a patient has no joints with active arthritis, no systemic manifestations attributable to JIA, no evidence of active uveitis, normal acute-phase reactants, and a physician global assessment indicating no disease activity. The state of ID by both JADAS10 and cJADAS10 criteria is established if the score is ≤ 1 in either oligoarthritis and polyarthritis.

Low (or minimal) disease activity (LDA) was defined by JADAS10 and cJADAS10 criteria [40, 42]. By these criteria, LDA is established when the JADAS10 and cJADAS10 are comprised between 1.1 and 2.0 and between 1.1 and 1.5, respectively, in oligoarthritis, and between 1.1 and 3.8 and between 1.1 and 2.5, respectively, in polyarthritis [43].

Assessment of JADAS was not included in our previous study [11] because this tool was developed after its publication. However, we could calculate both JADAS10 and cJADAS10 for all patients included in that study because all individual items were available in the database.

### Assessment of disease damage

The amount of articular and extraarticular damage was assessed through the Juvenile Arthritis Damage Index (JADI) [44]. Briefly, the JADI is composed in two parts: one devoted to the assessment of articular damage (JADI-A), and one devoted to the assessment of extraarticular damage (JADI-E). In the JADI-A, 36 joints or joint groups are assessed for the presence of damage, and the damage observed in each joint is scored on a 3-point scale (where 0 = no damage, 1 = moderate damage, and 2 = severe damage, ankylosis, or prosthesis). The maximum total score is 72. The JADI-E includes 13 items in 5 different organs/systems. Each item is scored as 0 if damage is absent or as 1 if damage is present. Due to the relevant impact of ocular damage on the child’s health, in each eye a score of 2 is given in case the patient has had ocular surgery, and a score of 3 is given in case the patient has developed legal blindness. The maximum total score is 17.

### Statistical analysis

Descriptive statistics are reported as median and interquartile range (IQR) for continuous variables and as absolute frequency and percentage for categorical variables. Comparisons of disease characteristics between patient groups were performed by means of the Mann-Whitney *U* test in the case of quantitative data and by means of the chi-square test, or Fisher’s exact test, as appropriate, in the case of categorical data. To search for factors associated with development of disease damage, we performed a multivariable logistic regression analysis, including the demographic and clinical features of both patient cohorts as explanatory variables and the presence of articular or extraarticular damage (i.e., JADI-A or JADI-E > 0) as an outcome variable.

The statistical packages were SAS 9.3 (Institute Inc., Cary, NC, USA) and Statistica (version 8.0, StatSoft Corp., Tulsa, OK, USA).

## Results

The demographic and clinical features of the 239 patients included in the MTX era cohort and of the 269 patients included in the biologic era cohort are presented in Table 1. Of the 310 patients enrolled in our previous study [11], 239 were placed in the MTX era cohort and 71 in the biologic era cohort as per the above criteria. The two cohorts were comparable for the male-to-female ratio and the frequency of antinuclear antibody positivity. Compared with the MTX era cohort, the biologic era cohort had, on average, a younger onset age and a longer disease duration at cross-sectional visit and included a lower proportion of patients with systemic and psoriatic arthritis and a greater proportion of patients with oligoarthritis. Because the disproportionate distribution of ILAR categories could affect the outcome figures, we assessed all study outcomes by dividing patients in the two “functional phenotypes” of oligoarthritis and polyarthritis. Patients were classified as having oligoarthritis or polyarthritis if they had involvement of 4 or less joints or 5 or more joints, respectively, in the whole disease course, irrespective of their individual ILAR category.

**Table 1:** Clinical characteristics at cross-sectional visit of patients in MTX and biologic era cohorts

	MTX era (*n* = 239)	Biologic era (*n* = 269)	*P*
Female	189 (79.1)	219 (81.4)	0.51
Median (IQR) age at disease onset, years	3.1 (1.8–5.8)	2.5 (1.6–4.7)	0.016
Median (IQR) disease duration at study visit, years	7.7 (6.1–10.2)	6.9 (5.4–9.7)	0.0005
Median (IQR) disease duration at first observation, years	0.7 (0.2–2.0)	0.6 (0.2–1.8)	0.52
ILAR category			
Systemic arthritis	23 (9.6)	17 (6.3)	0.0025
RF-negative polyarthritis	49 (20.5)	51 (19.0)	
RF-positive polyarthritis	5 (2.1)	0.0 (0.0)	
Persistent oligoarthritis	73 (30.5)	119 (44.2)	
Extended oligoarthritis	55 (23.0)	63 (23.4)	
Psoriatic arthritis	15 (6.3)	7 (2.6)	
Undifferentiated arthritis	19 (7.9)	12 (4.5)	
Patients with positive ANA	184 (77.0)	221 (82.2)	0.05
Past treatment			
Methotrexate	153 (64.0)	209 (77.7)	0.0007
Cyclosporin A	58 (24.3)	19 (7.0)	< 0.0001
Sulfasalazine	17 (7.1)	7 (2.6)	0.017
Biologic DMARDs	26 (10.9)	99 (36.8)	< 0.0001
Etanercept	23 (9.6)	84 (31.2)	< 0.0001
Infliximab	5 (2.1)	4 (1.5)	0.74
Adalimumab	–	27 (10.0)	–
Anakinra	–	7 (2.6)	–
Abatacept	–	3 (1.1)	–
Tocilizumab	–	4 (1.5)	–
Systemic glucocorticoids	92 (38.5)	85 (31.6)	0.10
Intraarticular glucocorticoids	187 (78.2)	250 (93.0)	< 0.0001
Present treatment			
Methotrexate	90/200 (45.0)	95/266 (35.7)	0.043
Cyclosporin A	18/200 (9.0)	3/266 (1.1)	< 0.0001
Biologic DMARDs	16/200 (8.0)	89/266 (33.5)	< 0.0001
Systemic glucocorticoids	8/200 (4.0)	8/266 (3.0)	0.56
No therapy	52/200 (26.0)	90/266 (33.8)	0.069

Data are number positive/number with information available (percentage) unless otherwise indicated; reference [11]

*MTX* methotrexate, *IQR* interquartile range, *RF* rheumatoid factor, *ANA* antinuclear antibodies, *DMARDs* disease antirheumatic drugs

The medications administered before cross-sectional visit and at the time of the visit are listed in Table 1. As expected, the biologic era cohort had received much more frequently biologic DMARDs than the MTX era cohort. Use of systemic glucocorticoids was equal in the two cohorts, whereas MTX and intraarticular glucocorticoids were given more commonly to patients in the biologic era cohort. At cross-sectional visit, more patients in the MTX era cohort were receiving MTX, whereas more patients in the biologic era cohort were on biologic DMARDs. Very few patients in both cohorts were taking systemic glucocorticoids. The percentage of patients who were given a biologic DMARD in the past and had been switched to a different biologic DMARD for inefficacy or intolerance was 22.2% (22/99) in the biologic era cohort and 7.7% (2/26) in the MTX era cohort. The comparison of disease activity states at cross-sectional visit between the two cohorts is shown in Table 2. Compared with patients in the MTX era cohort, patients in the biologic era cohort with either oligoarthritis or polyarthritis had consistently higher frequencies of ID and LDA by all criteria. Similar findings were seen for the comparison of the individual physician-centered and parent-reported outcomes, whose values were more commonly at the zero end of the scale in the biologic era cohort. Depending on the definition used, the proportion of patients with ID who were receiving no treatment was 41.7–42.9% and 28.6–31.6% in the oligoarthritis and polyarthritis category, respectively, in the MTX era cohort, and 36.6–50.8% and 25–42.6% in the oligoarthritis and polyarthritis category, respectively, in the biologic era cohort.

**Table 2:** Frequency of clinical inactive disease and low disease activity parameters at cross-sectional visit of patients in MTX and biologic era cohorts

	MTX era (*n* = 239)	Biologic era (*n* = 269)	*P*
Oligoarthritis	(*n* = 102)	(*n* = 136)	
Patients with physician global assessment = 0	34/100 (34.0)	85/134 (63.4)	< 0.0001
Patients with active joint count = 0	33/102 (32.4)	90/136 (66.2)	< 0.0001
Patients with parent global assessment = 0	37/96 (38.5)	61/114 (53.5)	0.0303
Patients with parent pain assessment = 0	39/95 (41.1)	65/122 (53.3)	0.074
Patients with ID by Wallace criteria	26/93 (28.0)	54/94 (57.4)	< 0.0001
Patients with ID by JADAS10	24/85 (28.2)	41/78 (52.6)	0.0015
Patients with ID by cJADAS10	28/95 (29.5)	63/111 (56.8)	< 0.0001
Patients with LDA by JADAS10	29/85 (34.1)	43/78 (55.1)	0.0070
Patients with LDA by cJADAS10	32/95 (33.7)	64/111 (57.7)	0.0010
Polyarthritis	(*n* = 137)	(*n* = 133)	
Patients with physician global assessment = 0	25/132 (18.9)	76/133 (57.1)	< 0.0001
Patients with active joint count = 0	25/136 (18.4)	78/133 (58.6)	< 0.0001
Patients with parent global assessment = 0	33/127 (26.0)	53/113 (46.9)	0.0007
Patients with parent pain assessment = 0	38/125 (30.4)	58/118 (49.2)	0.0028
Patients with ID by Wallace criteria	19/129 (14.7)	48/98 (49.0)	< 0.0001
Patients with ID by JADAS10	19/115 (16.5)	34/84 (40.5)	0.0002
Patients with ID by cJADAS10	21/122 (17.2)	54/112 (48.2)	< 0.0001
Patients with LDA by JADAS10	33/115 (28.7)	52/84 (61.9)	< 0.0001
Patients with LDA by cJADAS10	29/122 (23.8)	60/112 (53.6)	< 0.0001

Data are number positive/number with information available (percentage); reference [11]

*MTX* methotrexate, *ID* inactive disease, *LDA* low disease activity, *JADAS10* Juvenile Disease Activity Score 10, *cJADAS* Clinical Juvenile Disease Activity Score 10

The measurement of disease damage at cross-sectional visit revealed that the frequency of impairment of > 1 JADI-A items was higher in the MTX era cohort than in the biologic era cohort (18/102, 17.6% versus 15/136, 11% in oligoarthritis and 72/137, 52.6% versus 29/133, 21.8% in polyarthritis). Likewise, the frequency of involvement of > 1 JADI-E items was higher in the MTX era cohort than in the biologic era cohort (27/102, 26.5% versus 22/136, 16.2% in oligoarthritis and 43/137, 31.4% versus 18/133, 13.5% in polyarthritis). Most individual JADI items impaired in the MTX era cohort decreased in frequency in the biologic era cohort, with reduction in ocular damage in oligoarthritis being most notable. The sole JADI items that were detected in more than 5% of patients in the biologic era cohort were temporomandibular damage in oligoarthritis and polyarthritis, ankle damage in polyarthritis, and leg-length inequality in oligoarthritis (Table 3).

**Table 3:** Percentages of Juvenile Arthritis Damage Index (JADI) articular and extraarticular items by therapeutic era in patients with oligoarthritis and polyarthritis

	Oligoarthritis (*n* = 238)		Polyarthritis(*n* = 270)	
	MTX era (*n* = 102)	Biologic era (*n* = 136)	MTX era (*n* = 137)	Biologic era (*n* = 133)
Articular				
Temporomandibular	7 (6.9)	10 (7.4)	16 (11.7)	13 (9.8)
Cervical spine	2 (2.0)	1 (0.7)	13 (9.5)	2 (1.5)
Shoulder	1 (1.0)	0 (0.0)	14 (10.2)	1 (0.8)
Elbow	3 (2.9)	1 (0.7)	27 (19.7)	1 (0.8)
Wrist	2 (2.0)	0 (0.0)	31 (22.6)	2 (1.5)
Metacarpophalangeal	1 (1.0)	1 (0.7)	15 (10.9)	3 (2.3)
Proximal interphalangeal	2 (2.0)	0 (0.0)	29 (21.2)	5 (3.8)
Hip	1 (1.0)	2 (1.5)	17 (12.4)	1 (0.8)
Knee	3 (2.9)	1 (0.7)	19 (13.9)	4 (3.0)
Ankle	5 (4.9)	2 (1.5)	13 (9.5)	8 (6.0)
Metatarsophalangeal	0 (0.0)	0 (0.0)	20 (14.6)	2 (1.5)
Extraarticular				
Ocular	13 (12.7)	1 (0.7)	8 (5.8)	6 (4.5)
Muscular atrophy	6 (5.9)	6 (4.4)	13 (9.5)	2 (1.5)
Osteoporosis	1 (1.0)	0 (0.0)	2 (1.5)	1 (0.8)
Avascular necrosis	0 (0.0)	0 (0.0)	0 (0.0)	1 (0.8)
Scoliosis	6 (5.9)	4 (2.9)	11 (8.0)	1 (0.8)
Leg length inequality	9 (8.8)	15 (11.0)	9 (6.6)	6 (4.5)
Striae rubrae	0 (0.0)	0 (0.0)	3 (2.2)	1 (0.8)
Subcutaneous atrophy	4 (3.9)	1 (0.7)	5 (3.6)	5 (3.8)
Growth failure	2 (2.0)	0 (0.0)	17 (12.4)	1 (0.8)
Pubertal delay	0 (0.0)	0 (0.0)	5 (3.6)	0 (0.0)
Diabetes	0 (0.0)	0 (0.0)	0 (0.0)	0 (0.0)
Amyloidosis	0 (0.0)	0 (0.0)	0 (0.0)	0 (0.0)
Others	1 (1.0)	0 (0.0)	0 (0.0)	0 (0.0)

Data are number positive/number with information available (percentage) unless otherwise indicated; reference [11]

**Fig. 1:**
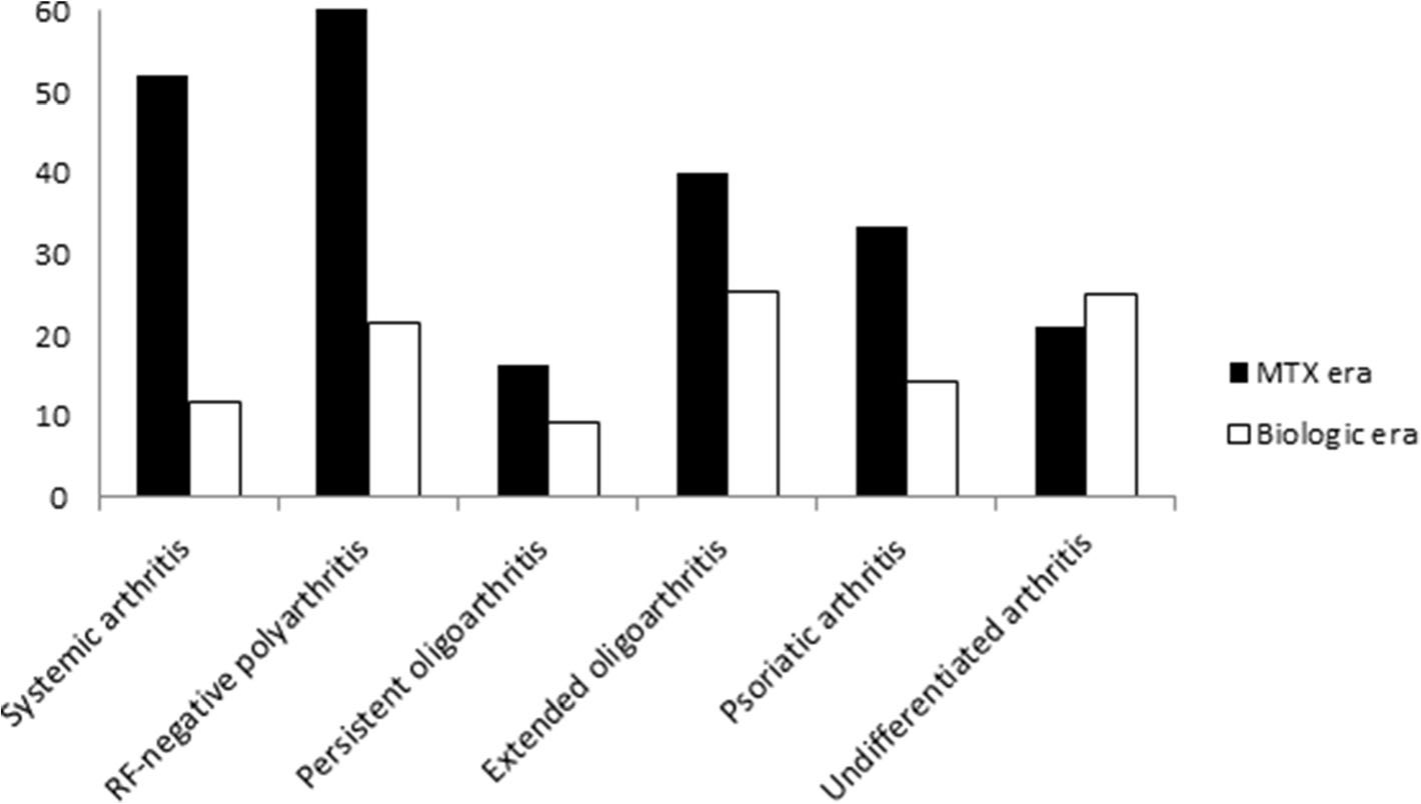
Percentage of patients with articular damage (i.e., JADI-A > 0) by JIA category in the two study cohorts

**Fig. 2:**
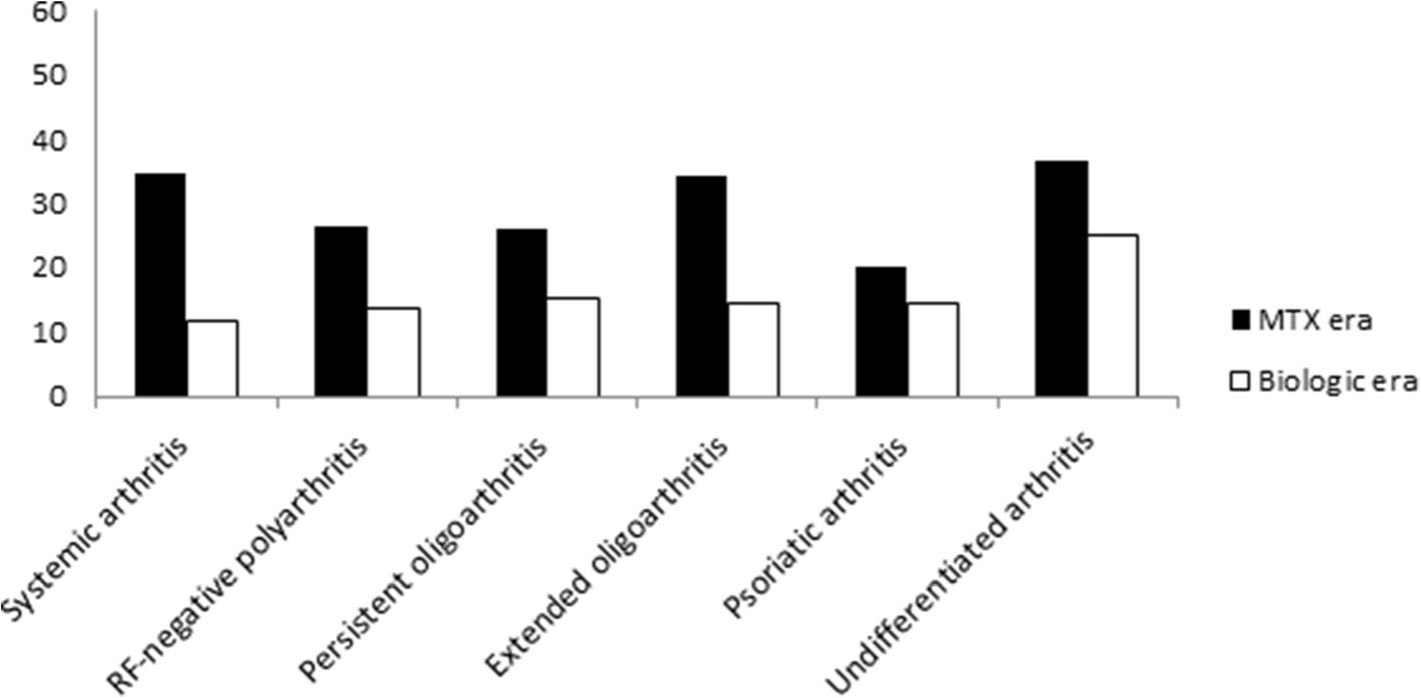
Percentage of patients with extraarticular damage (i.e., JADI-E > 0) by JIA category in the two study cohorts

**Fig. 3:**
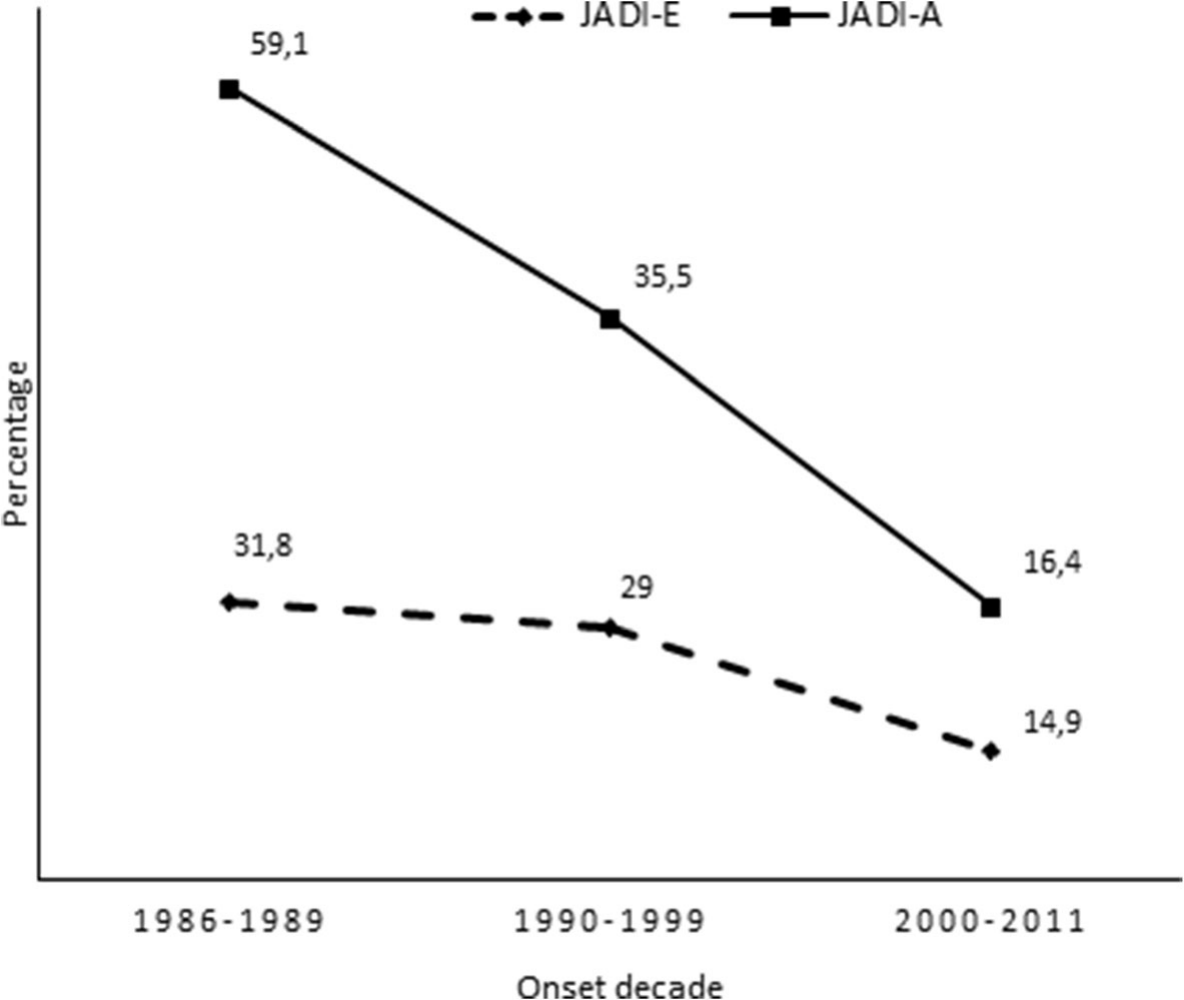
Percentage of patients with articular damage (i.e., JADI-A > 0) and extraarticular damage (i.e., JADI-E > 0) over 25 years in the study patients divided by decade of disease onset

The lower frequency of articular and extraarticular damage in the biologic era cohort was confirmed by assessment by ILAR category (Figs. 1 and 2). To examine the temporal trend of damage development over the 25 years of our analysis, we divided the study patients into three groups by decade of disease onset (1986–1989, 1990–1999, 2000–2011). This evaluation, which is depicted in Fig. 3, emphasized the marked decrease in damage over time and the more pronounced decline in the biologic era.

On multivariable logistic regression analysis, articular damage was found to be more likely in the MTX era cohort (versus the biologic era cohort; odds ratio (OR) 2.68, 95% confidence interval (CI) 1.72–4.15) and in patients with polyarthritis (versus patients with oligoarthritis; OR 3.75, 95% CI 2.36–5.96), with older age at disease onset (OR 1.07, 95% CI 1.0–1.14), and with longer disease duration at study visit (OR 1.11, 95% CI 1.04–1.19). Extraarticular damage was more likely in the MTX era cohort (versus the biologic era cohort; OR 2.52, 95% CI 1.62–3.92) and in patients with older age at disease onset (OR 0.91, 95% CI 0.84–0.98).

The frequency of inactive disease and articular and extraarticular damage in the 71 patients from our previous study [11] diagnosed after 1999 and in the 198 patients in the more recent cohort was comparable, which supported their combination in a single group (result not shown).

## Discussion

We compared the rate of ID and LDA and the frequency and distribution of articular and extraarticular damage between two inception cohorts of JIA patients with long-standing disease (i.e., with a disease duration ≥ 5 years) who had their disease onset during the “MTX era” (i.e., between 1986 and 1999) or during the “biologic era” (i.e., after 2000). We found that the states of ID and LDA were achieved more frequently by patients in the biologic era cohort and that this cohort also had a lower frequency of cumulative damage in both articular and extraarticular domains than the MTX era cohort. These findings provide a demonstration that the recent therapeutic progresses have improved markedly the outlook of children with JIA as compared to the pre-biologic era.

As expected, patients in the biologic era cohort had received much more frequently biologic DMARDs than patients in the MTX era cohort. However, the former sample was also given more commonly MTX and intra-articular glucocorticoids, which reflects the current shift toward a more aggressive global therapeutic approach [45]. Systemic glucocorticoids were used in around 30% of patients in both cohorts, but at cross-sectional visit, only less than 4% of the patients were still taking these medications.

Our findings in the biologic era cohort indicate that more than 50% of patients with oligoarthritis and 40–50% of patients with polyarthritis treated with contemporary therapies may achieve ID over the long-term. A further 5–10% reached the state of LDA, which is regarded as a qualified and valid therapeutic goal, particularly in patients with long-standing disease [31]. These figures are not easily comparable with those reported in other recent surveys because of differences in proportion of disease categories, length of follow-up, outcome endpoints, treatments, and statistical methodology. In a multicenter Canadian study of 1104 children with JIA, Guzman and co-workers [46] found that the cumulative probability of attaining disease remission within 5 years was 46–57% across JIA categories, except for rheumatoid factor (RF)-positive and RF-negative polyarthritis, whose probability was 0% and 14%, respectively. Of a British cohort of 1415 children and adolescents with JIA, between 48% and 61% achieved MDA and between 25% and 38% achieved ID, depending on the criteria used, at 1 year following presentation [47]. A German multicenter study of 695 JIA patients found that at the 12-month follow-up 40% of them had achieved a continuously ID for at least 3 months [48]. Boiu et al. [49] reported that 31% and 56% of 95 patients with a median disease duration of 3.5 years had ID and LDA, respectively.

The improvement in disease prognosis from the MTX to the biologic era was also underscored by the marked decrease over time in the frequency of articular and extraarticular damage. Most of the JADI items that were impaired in the MTX era cohort were less frequently affected in the biologic era cohort, with the most notable improvement being the reduction in ocular sequelae in the oligoarthritis group. There were, however, some forms of damage whose impact was not diminished in the biologic era cohort, which included temporomandibular and ankle alterations and leg-length inequality. The unchanged prevalence of temporomandibular joint damage may depend on the frequent paucity of symptoms in temporomandibular joint arthritis, which may lead to delayed diagnosis and late institution of appropriate therapy [50]. The ankle is a complex anatomic area, which comprises several individual joints (tibio-crural, subtalar, and intertarsal) and a number of tendons, all of which can become inflamed. For this reason, complete control of ankle arthritis may prove difficult. Notably, ankle involvement has been found to be associated with poorer prognosis and with a lesser response to intra-articular glucocorticoid therapy in JIA [51, 52]. The continued occurrence of leg-length discrepancy, which is a well-known consequence of knee monoarthritis, emphasizes the need of timely intra-articular glucocorticoid injection in this joint, a procedure that may prevent this complication [53].

Our analysis should be interpreted in the light of some caveats. Because the study was conducted in Western pediatric rheumatology centers, its results may not be generalizable, particularly to geographic settings where the costly biologic medications may not be available or affordable [54]. We focused on disease-centered measures of disease activity and damage and did not assess physical function and health-related quality of life. Studies in recent series have shown that the majority of JIA patients have normal or near-normal functional ability and achieve a health-related quality of life that is similar to that of healthy peers [49, 55]. Outcome endpoints did not include imaging procedures, which could provide a more objective estimation than clinical tools. We only examined the rate of ID at a single point in time and did not evaluate its duration, which could have allowed us to calculate the rate of clinical remission on and off medications [56]. The inability to calculate the percentage of patients who failed the first DMARD did not allow us to assess the time spent before the achievement disease control, which may influence the prognosis. In addition, we could not evaluate the proportion of patients who achieved remission in less than 5 years and were, then, lost to follow-up. The differences in the frequency of disease activity and damage between the two cohorts could have been affected by the disparity in age at onset, disease duration, and proportion of JIA categories. Some patients in the MTX era cohort had received biologic DMARDs, which could represent a confounding factor. However, it is likely that most of these patients had started biologics later and were given an overall less aggressive treatment than patients in the biologic era cohort, in line with the aforementioned shift in the therapeutic paradigm between the two eras. We should finally acknowledge that there were no patients with RF-positive polyarthritis in the biologic era cohort and that only 2% patients in the MTX era sample had this disease category. The low proportion of patients with RF-positive polyarthritis, which is one of the most severe forms of JIA, could have influenced the results.

In conclusion, our study provides evidence from standard clinical care of the remarkable prognostic improvement obtained with the recent therapeutic advance in JIA. However, there is still a sizeable proportion of patients who do not achieve complete disease quiescence with contemporary therapies. In addition, some important sources of damage, such as temporomandibular and ankle joint damage and leg-length inequality, remain prevalent. These findings underscore the need for newer treatments and treatment strategies that have the ability to better control disease activity in the most resistant cases and to further reduce the development of disease damage.

## Data Availability

Patients in the MTX era cohort and part of the patients in the biologic era cohort were taken from a previous cross-sectional study published by our group [11].

## Abbreviations

ANA: Antinuclear antibodies
CRP: C-reactive protein
DMARDs: Disease-modifying antirheumatic drugs
ESR: Erythrocyte sedimentation rate
ID: Inactive disease
ILAR: International League of Associations for Rheumatology
JADAS: Juvenile Arthritis Disease Activity Score
JADI-A: Articular Juvenile Arthritis Damage Index
JADI-E: Extraarticular Juvenile Arthritis Damage Index
JIA: Juvenile idiopathic arthritis
LDA: Low disease activity
MTX: Methotrexate
PRINTO: Paediatric Rheumatology INternational Trials Organization
RCT: Randomized controlled trials
RF: Rheumatoid factor
TNF: Tumor necrosis factor
VAS: Visual analog scale
